# Supplementary material for: Genome-Wide SNPs and InDels Characteristics of Three Chinese Cattle Breeds
Source: Animals (Basel). 2019 Aug 22;9(9):596. doi: 10.3390/ani9090596 (PMC6769757; doi:10.3390/ani9090596)
Supplement: Supplementary file 1 [file animals-09-00596-s001.zip › Figure_S1.docx]

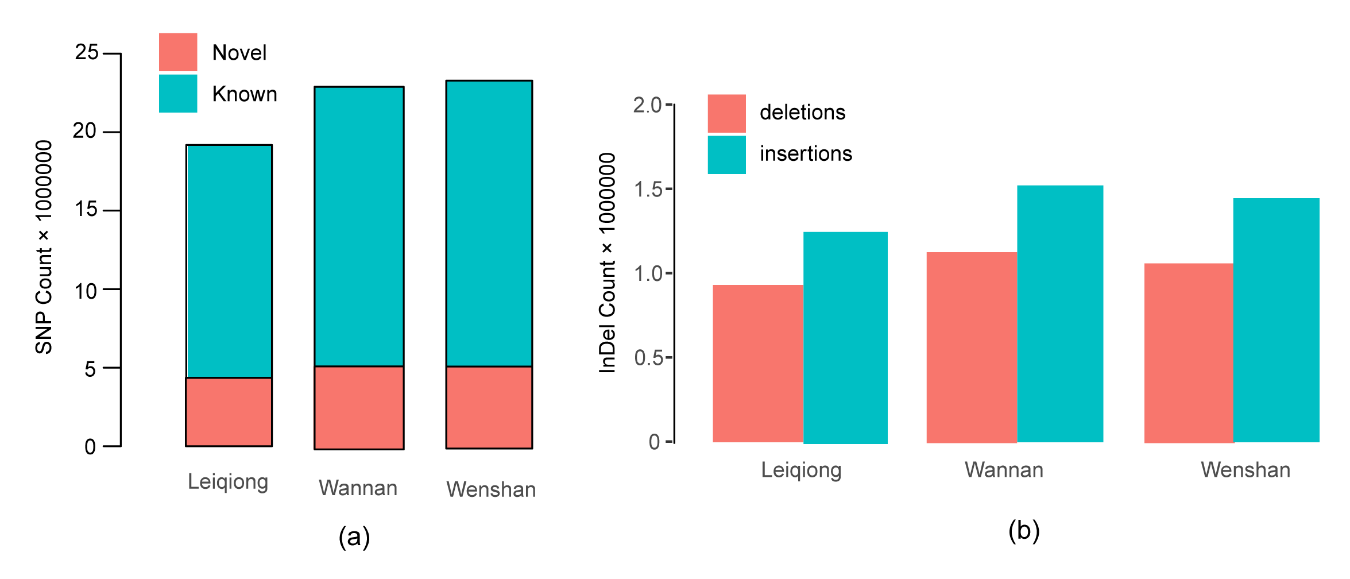


**Figure S1** Total/novel SNPs count and deletions/insertions count in three breeds of Chinese indicine. **(a)** The identified novel and known SNPs for each breed. **(b)** The identified deletions and insertions for each breed
